# The COVID-19 pandemic and access to vaccines in Latin America and the Caribbean: strategies adopted by the WHO and PAHO

**DOI:** 10.1590/0102-311XEN016225

**Published:** 2026-08-03

**Authors:** Luana Bermudez, Cristiani Vieira Machado

**Affiliations:** 1 Escola Nacional de Saúde Pública Sergio Arouca, Fundação Oswaldo Cruz, Fiocruz,Rio de Janeiro, Brasil.

**Keywords:** Pandemia de COVID-19, Acesso a Medicamentos Essenciais e Tecnologias em Saúde, Vacinas, América Latina, COVID-19 Pandemic, Access to Essential Medicines and Health Technologies, Vaccines, Latin America, Pandemia de COVID-19, Acceso a Medicamentos Esenciales y Tecnologías Sanitarias, Vacunas, América Latina

## Abstract

This study analyzes the strategies of the World Health Organization (WHO) and the Pan American Health Organization (PAHO) to promote access to COVID-19 vaccines in Latin America and the Caribbean, exploring the vaccines administered, vaccination coverage in the countries, and inequalities in vaccination in the region. The study method combines analysis of secondary data and of documents, and interviews with key informants. The results show advances and limitations of the WHO and PAHO initiatives to promote vaccine equity, adopt redistributive measures to reduce inequalities in access to vaccines, and achieve the established global coverage goals. This study identified structural and political-institutional factors that favored or hindered the action of these organizations in vaccine distribution and promotion of vaccination equity during the pandemic. The role of these multilateral organizations in global and regional health arrangements is critically discussed in terms of their legitimacy, institutional capacity, and technical competence. The conclusions highlight the importance of strengthening mechanisms of global solidarity and helping Latin American nations overcome structural problems, such as limited national production and scientific and technological dependence, which hampered vaccination processes and aggravated inequalities between countries during the health emergency.

## Resumen

El artículo analiza las estrategias de la Organización Mundial de la Salud (OMS) y la Organización Panamericana de la Salud (OPS) para promover el acceso a la vacunación contra la COVID-19 en América Latina y el Caribe, explorando las vacunas administradas, la cobertura vacunal en los países y las desigualdades en la vacunación en la región. La metodología combina el análisis de datos secundarios, documentos y entrevistas con informantes clave. Los resultados apuntan a avances y limitaciones de las iniciativas de la OMS y la OPS para promover la equidad en la vacunación, adoptar medidas redistributivas para reducir las desigualdades en el acceso a las vacunas y alcanzar los objetivos globales de cobertura establecidos. Se identifican factores estructurales y político-institucionales que favorecieron o dificultaron la actuación de estas agencias en la distribución de vacunas y la promoción de la equidad en la vacunación durante la pandemia. Se discute críticamente el papel de estas organizaciones multilaterales en los acuerdos globales y regionales de salud, a la luz de su legitimidad, capacidad institucional y competencia técnica. Las conclusiones destacan la necesidad de fortalecer los mecanismos de solidaridad global y apoyar a las naciones latinoamericanas para superar problemas estructurales, como la producción nacional limitada y la dependencia científica y tecnológica, que dificultaron los procesos de vacunación y agravaron las desigualdades entre países durante la emergencia sanitaria.

## Resumo

O artigo analisa as estratégias da Organização Mundial da Saúde (OMS) e da Organização Pan-Americana da Saúde (OPAS) para promover o acesso à vacinação contra a COVID-19 na América Latina e no Caribe, explorando as vacinas administradas, a cobertura vacinal nos países e as desigualdades na vacinação na região. A metodologia combina análise de dados secundários, de documentos e entrevistas com informantes-chave. Os resultados apontam avanços e limitações das iniciativas da OMS e da OPAS para promover a equidade vacinal, adotar medidas redistributivas para reduzir as desigualdades no acesso às vacinas e alcançar as metas globais de cobertura estabelecidas. Identificam-se fatores estruturais e político-institucionais que favoreceram ou dificultaram a atuação dessas agências na distribuição de vacinas e na promoção da equidade na vacinação durante a pandemia. Discute-se criticamente o papel dessas organizações multilaterais nos arranjos globais e regionais de saúde, à luz de sua legitimidade, capacidade institucional e competência técnica. As conclusões ressaltam a necessidade de fortalecer mecanismos de solidariedade global e de apoiar as nações latino-americanas na superação de problemas estruturais, como a produção nacional limitada e a dependência científica e tecnológica, que dificultaram os processos de vacinação e exacerbaram as desigualdades entre países durante a emergência sanitária.

## Introduction

The COVID-19 pandemic intensified social inequalities in Latin America and the Caribbean as it impacted a region already marked by low economic growth, insufficient investment in public policies, and political instability ^1^ ^,^ ^2^. The weak regional economic performance before the crisis, with an average annual growth of 0.3% between 2014 and 2019 ^3^, was followed by an economic contraction in 2020, with a 6.8% reduction in gross domestic product (GDP), the worst among developing regions ^4^. In the second half of 2021, inflation reached 6.6%, a level not seen since October 2008 ^5^, while unemployment increased between 2019 and 2022, aggravating the crisis. The mortality rate increased, life expectancy at birth decreased, and total and per capita GDP declined between 2020 and 2022, reflecting the deep impact on regional economic and social dynamics.

The region also reported significant excess mortality, an indicator that covers deaths caused by the coronavirus and other causes in the context of the pandemic. In 2020 and 2021, 2,273,620 additional deaths were reported when compared to the predicted number, based on averages from previous years ^6^, corresponding to 15% of the excess deaths in the world ^7^, despite the region being home to only 8% of the global population. Of the 10 countries with the highest percentage of excess deaths in the world in 2020, 9 were in Latin America ^8^ ^,^ ^9^.

The 2020 United Nations General Assembly highlighted the importance of global solidarity to address the COVID-19 pandemic and international cooperation to ensure access to medicines, vaccines, and medical equipment ^10^ ^,^ ^11^, and the World Health Assembly ^12^ approved a resolution recommending “*universal, timely and equitable access to, and fair distribution of, all quality, safe, efficacious and affordable essential health technologies and products* (...) *that are required in the response to the COVID-19 pandemic as a global priority*” ^130^.

The World Health Organization (WHO), created in 1948 to be the global health authority ^14^ ^,^ ^15^, sought to take the lead in the response to the COVID-19 pandemic, relying on its regional representations, including the Pan American Health Organization (PAHO), created in 1902, one of the oldest multilateral cooperation initiatives in the world ^16^. PAHO has two institutional roles: it acts as a regional office of WHO and as a specialized agency of the Inter-American System. PAHO plays an essential role in the regional response to health emergencies by providing technical support and coordination of efforts among countries.

These organizations have faced a crisis marked by loss of protagonism, funding shortages, fragmented governance, and criticism of their ability to respond to health emergencies. New actors in the health field have increased competition and fragmentation, intensifying questions about their effectiveness and credibility. Despite the challenges and disapproval, which became worse during the pandemic, WHO remains the only organization with the legitimacy to lead global health ^17^ ^,^ ^18^, and PAHO remains and agency of fundamental importance at the regional level.

In a scenario of global asymmetries in economic, scientific, and technological development, in the face of health emergencies, the differences between those who have access to health products and services and those who do not enhances inequities among peoples, which can lead to humanitarian crises. The increasing concentration of productive and innovative capacity and monopolization in the health sector, without adequate international mechanisms to promote equity and redistribution among countries, make it impossible to achieve universal health ^19^.

Many determinants of health conditions and inequalities are beyond the reach of national governments and the health sector, requiring articulated policy solutions on a global scale ^20^. However, the COVID-19 pandemic demonstrated that the innovation architecture is not structured to guarantee universal access to biomedical products, as the intellectual property system favors covert competition, monopolies, and practices that deepen the dependence of low-income countries ^21^. Therefore, the challenges to ensuring equitable access to the benefits of biomedical research & development (R&D) reinforce the need to readjust this system towards the public interest ^22^. This process requires strategies to strengthen scientific and industrial capacities in all regions, as well as rules to ensure the global sharing of knowledge and technology. The expansion of regional production hubs is an essential step to address current crises and prepare the world for future health emergencies ^23^.

WHO established a global target to vaccinate 70% of all populations against COVID-19 by mid-2022 to control the pandemic. The development and the timely and equitable access to safe and effective vaccines would be crucial to reducing severe cases and deaths and achieving the end of the COVID-19 pandemic. In this context, PAHO supported its member countries in their pandemic responses. Despite different national strategies, all countries implemented vaccination measures.

Given the international relevance of these organizations and the importance of access to strategic supplies and vaccination for public health, it is important to explore how the WHO and PAHO have acted to guarantee access to COVID-19 vaccines and reduce inequalities in vaccination among countries. Given the recent nature of the pandemic, there is still a relative scarcity of studies on the subject.

This study aims to analyze the strategies of the WHO and PAHO to promote access to the COVID-19 vaccines. It also describes the vaccines administered and vaccination coverage in Latin American and Caribbean countries, while considering vaccination inequalities.

## Method

This is a descriptive exploratory study focused on the actions of WHO and PAHO to promote access to COVID-19 vaccines, guided by four pillars of analysis:

a) Socioeconomic and epidemiological context of Latin American and Caribbean countries from 2019 to 2023;

b) Strategies to promote access to COVID-19 vaccines adopted by the WHO and PAHO;

c) Vaccination coverage and vaccines used in the region;

d) Determinants and challenges of inequalities in access to vaccines in the region.

The study was based on secondary data and document analysis, complemented by interviews with key informants.

Secondary data were collected from different sources: socioeconomic data from the statistical yearbooks of the Economic Commission for Latin America and the Caribbean (ECLAC) of 2019 to 2023 ^24^, epidemiological data from the Our World in Data ^25^ platform, the WHO database on excess mortality associated with COVID-19 ^7^, the Financial Times repository for excess mortality of 2020 to 2021 ^8^, and PAHO dashboards on vaccination and COVAX deliveries ^26^ ^,^ ^27^.

Official documents, reports, and press releases available on the websites of WHO (https://www.who.int), PAHO (https://www.paho.org), and ECLAC (https://www.cepal.org) related to the pandemic, international cooperation initiatives, and regional strategies to promote access to COVID-19 vaccines were analyzed. The study period was from 2020 to 2023, covering from the beginning of the pandemic until the consolidation of vaccination strategies. Different sources were considered, such as technical notes, reports, press releases, and plans related to international cooperation and access to COVID-19 vaccines.

In addition, six semi-structured interviews were conducted with key informants on the strategies of WHO and PAHO. The selection criteria for the interviewees were: vaccine expertise and participation in initiatives, councils or expert groups, such as the Coalition for Epidemic Preparedness Innovations (CEPI), GAVI, the Vaccine Alliance (Gavi), and the Technical Advisory Group (TAG) of the COVID-19 Technology Access Pool (C-TAP).

Six interviewees (three from WHO, two from PAHO, and one from CEPI) were invited to participate in the study via email, with the objectives clearly explained. The interviews were conducted in person (1) and online (5), according to the interviewee’s availability. Each interview lasted about one hour, and all of them were recorded and transcribed by the researcher. Content analysis was performed according to the study pillars, without using any software tool. In the presentation of the results, confidentiality was ensured using the letter “E” followed by a number.

Also, a bibliographic search was conducted to identify publications from 2020 to 2022 on access to COVID-19 vaccines. Searches were performed on the VHL Portal, SCOPUS, and Web of Science using Health Sciences Descriptors (DeCS, acronym in Portuguese) related to the topic (Supplementary Material: Appendix 1; https://cadernos.ensp.fiocruz.br/static//arquivo/suppl-e00016225-ing_5939.pdf), and found 51, 64, and 10 articles respectively. Of these, 34 were selected for further review.

The study project was approved by the Research Ethics Committee of the Sergio Arouca National School of Public Health, Oswaldo Cruz Foundation (ENSP/FIOCRUZ, CAAE 60435522.6.0000.5240).

## Results

### Vaccine access strategies adopted by WHO and PAHO

WHO’s main strategy was ACT-A (Access to COVID-19 Tools Accelerator), a global consortium launched in 2020 involving more than 40 countries for the development, production, and access to diagnostics, therapeutics, and vaccines against COVID-19 ^28^.

Under the ACT-A, the COVAX (COVID-19 Vaccines Global Access) mechanism was launched to accelerate the development, acquisition, and distribution of vaccines, coordinated by CEPI, Gavi and WHO, in collaboration with United Nations Children’s Fund (UNICEF). CEPI led R&D activities, Gavi managed the large-scale purchase and distribution; WHO provided guidance on vaccine policies, regulation, safety, R&D, allocation, preparation, and delivery in countries. UNICEF supported vaccine acquisition, transport and logistics ^29^.

The goal of COVAX was to make two billion doses of vaccines available by December 2021, based on the global number of high-risk individuals and frontline healthcare workers ^30^. The mechanism had two forms of participation: countries could participate in COVAX advance Market Commitment (AMC), funded by donations, or self-finance the purchase of doses to vaccinate 10% to 50% of their populations ^30^. By August 2020, 172 countries, representing more than 70% of the world’s population, had joined the mechanism 80 through self-financing and 92 through COVAX AMC ^31^.

Another strategy of WHO was C-TAP, which was launched in partnership with Costa Rica in 2020 ^32^, aiming to encourage product manufacturers to share their intellectual property and know-how, thereby facilitating production and reducing barriers to access ^33^.

Even though WHO was in favor of fair distribution through COVAX and technology sharing through C-TAP, it became clear in early 2021 that developing countries would face challenges in receiving COVID-19 vaccines. In 2021, WHO called for companies interested in hosting a messenger RNA (mRNA) technology transfer hub and, in June of that year, selected a South African consortium. WHO created the mRNA Technology Transfer Programme to develop sustainable production capacities in developing countries ^34^. While crucial for access to technologies, this initiative did not address the acute phase of the pandemic, whose immediate need was the rapid and equitable distribution of available vaccines. The program would be a long-term solution to strengthen local production capacity for the prevention of and response to future health emergencies.

In the Americas, PAHO played an instrumental role in implementing vaccine access initiatives during the pandemic − it encouraged vaccine donations to the region by negotiating vaccine sharing with governments, and supported COVAX operations, including the redistribution of vaccines among countries ^35^.

The goal of COVAX in the region was to provide vaccines for 20% of the population of each participating country by the end of 2021 ^36^. Since COVAX did not achieve the expected success in its first year, in August 2021, PAHO decided to use the Revolving Fund to acquire vaccines and complement the supply to its Member States ^37^. This Fund, created in 1979, is a vaccine acquisition mechanism that negotiates price reductions to 42 countries, ensuring the sustainability of national immunization programs ^38^.

In addition, PAHO called for regional companies to participate in the WHO mRNA Technology Transfer Programme ^39^. In 2021, it announced that Bio-Manguinhos/FIOCRUZ (Immunobiological Technology Institute, FIOCRUZ, Brazil) and Sinergium Biotech (Argentina) were selected to strengthen the production capacity in the region and reduce its dependence on imports in the face of health emergencies ^40^.

Also in 2021, PAHO proposed the Regional Platform for Advancing the Production of Vaccines and other Health Technologies for COVID-19 in the Americas, aiming to bring together public and private partners to boost R&D and the production of technologies, and promote collaboration between countries and organizations so they can explore existing biomanufacturing capacity ^41^. The PAHO selection of mRNA producers and the creation of the Regional Platform can impact future health crises by ensuring regional infrastructure for the production and distribution of vaccines and health technologies, reducing dependence on imports and promoting equity in access to health supplies.

PAHO also supported countries in receiving vaccines by planning vaccine demand, logistics, and cold chain management. It also helped them strengthen surveillance of adverse events and reporting systems, train healthcare professionals, generate vaccine demand, and provide guidelines for risk communication.

The strategies adopted by both organizations aimed to promote equity in access to COVID-19 vaccines, despite the challenges of global competition and limited production − topics that were addressed by the interviewees.

According to one interviewee (E1), several factors affected the allocation through COVAX, such as the interruption of vaccine shipments from India when the pandemic hit the country hard, and the actions of a European consortium for the joint purchase of vaccines. The bloc sued AstraZeneca for breach of contract and tried to force the delivery of 20 million doses from India to the consortium. Many pharmaceutical companies failed to meet delivery deadlines stipulated in the contracts.

These challenges were highlighted by another interviewee:

“*The biggest operational challenge was in the spring of 2021, when we couldn’t access vaccines from India. High-income countries bought all the vaccines, and the Serum Institute of India couldn’t export. GAVI didn’t have immediate access to funds for advance purchase agreements necessary to be at the front of the line*” (E6).

Some interviewees focused on challenges in the relationship of PAHO, WHO, and other organizations. Interviewee E1 mentioned that the inclusion of the Revolving Fund in COVAX faced resistance. However, since other regions did not have an equivalent fund, it was possible to include it as a vaccine acquisition mechanism for the Americas.

Interviewee E4 mentioned that the decision to include the Revolving Fund to complement the COVAX vaccine acquisition was made by the top management of WHO, given the delays and the tendency for Latin American countries to be at the back of the line to receive vaccines. The Revolving Fund could have been used since the beginning of the pandemic to ensure equitable vaccination coverage ^42^ because, by projecting a higher demand, it reduces the price of vaccines and increases the purchasing power of the States ^38^. One interviewee addressed the role of PAHO in the governance of COVAX:

“*We were one of the two vaccine acquisition agents.* (...) *an important role, but we should have had a bigger role. We participated in COVAX calls, but not at the highest level. We were invited for specific references to vaccine acquisition actions. We did not participate in AMC discussions with suppliers.* (...) *UNICEF had more access; we maintained our channels with WHO.* (...) *The team was involved in components of the discussion, but we did not have the authority to make decisions*” (E4).

The interviewee emphasized that the regional directors participated in weekly updates to WHO. For the interviewee, WHO paid less attention to Latin America and the Caribbean than to other regions, especially Africa. Then, the interviewee believes that PAHO could have done more:

“*The leadership at the time was focused on doing what we did, until the summer of 2021, when we opened the third component of our regional strategy. That was too late*” (E4).

According to PAHO ^42^, two factors affected its response capacity: the centralization of decisions at WHO and the dependence on COVAX to acquire and distribute vaccines. There were also internal factors, such as financial and human resource crises, as well as communication problems between PAHO headquarters and its country offices.

Another problem is that self-financed countries could purchase vaccines for up to 50% of their populations, while COVAX AMC countries could only receive vaccines for 20% of their population, which exacerbated inequalities ^42^. Not to mention the price variations: the Moderna vaccine in the United States cost USD 15, while in Argentina it cost 50% more ^3^.

Despite the challenges, E1 defended the integration of the Revolving Fund into COVAX because direct purchase by PAHO would increase global competition and vaccine shortages. The interviewee argued that COVAX helped countries that otherwise would not have access to vaccines.

Another interviewee (E5) highlighted that the Regional Platform promoted interaction between local producers, strengthening production and regional regulatory capacities. This is expected to have an impact in future crises.

It should be noted that, in addition to initiatives focused on vaccine distribution, WHO and PAHO adopted other actions to strengthen vaccination in countries, including the provision of training and organization of cold chain networks.

### Vaccination in Latin America and the Caribbean

Latin American and Caribbean countries gained access to vaccines through COVAX, donations, and bilateral negotiations for the purchase or transfer of technology. Most joined COVAX: 23 countries participated, 13 were self-financed, and 10 joined AMC ^42^.

Vaccination in the region began in December 2020 in Chile, Mexico, Costa Rica, and Argentina, and in January 2021 in Brazil ^43^. Latin American countries started receiving vaccines from COVAX in March 2021, in reduced quantities ^44^, totaling 77.8 million doses in 2021 ^45^ and 155 million doses by October 2022, representing 10% of the total vaccines in the region. Of these doses, 73% were provided by the Revolving Fund, while 27% were acquired by the countries. In addition, 32% of the vaccines were donated by countries or financed by COVAX AMC, while 68% were self-financed. Countries in the region received about 30% of the doses bought by COVAX ^42^.

The subregions that were most benefited by international donations were the Caribbean, Central America, and the Andean region, which received doses from developed countries ^5^.

**Figure 1:**
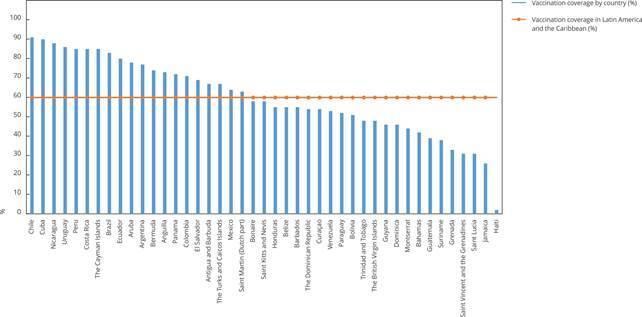
Vaccination coverage (% of population) by country and average for the Latin America and Caribbean region, from December 2020 to December 2022.

Source: prepared by the authors based on data from Our World in Data (Mathieu et al. ^25^). Note: percentage of the population with a complete initial vaccination schedule of two doses or a single dose.

Figure 1 shows asymmetries in vaccination. Chile, Cuba, and Nicaragua had the highest vaccination coverage, while Haiti, Jamaica, and Saint Lucia had the lowest. In December 2022, in most Latin American countries, more than 70% of the population was vaccinated. In the Caribbean, 4 countries had 70% of their populations vaccinated, and 7 had less than 40%, the target set by WHO for the end of 2021.

**Figure 2:**
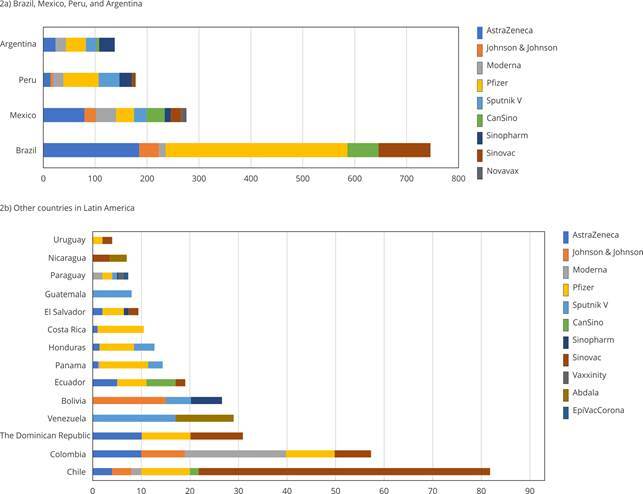
Vaccines acquired by Latin American countries, by manufacturer.

Source: prepared by the authors with data from Harrison et al. ^43^. Note: data were separated into two parts due to the scale of the graphs.

National strategies varied. More populous countries were among those that bought more than 100 million doses (Figure 2a), except for Colombia. Brazil prioritized local production, initially relying on advance purchase agreements and technology transfer with AstraZeneca (England) and Sinovac Biotech (China) ^46^ ^,^ ^47^ ^,^ ^48^. Later, the country bought vaccines from Pfizer (United States), Johnson & Johnson (Canada), CanSino (China), and Moderna (United States), showing supplier diversification while strengthening its national production capacity. Argentina and Mexico, on the other hand, opted for diversified suppliers and also made an agreement with AstraZeneca for technology transfer ^49^, but had problems in production. Peru bought the equivalent of five doses per person from various suppliers, predominantly Pfizer.

Figure 2b highlights Chile, with 20 million inhabitants, which bought more than 80 million doses, primarily from Sinovac, followed by Pfizer, AstraZeneca, Johnson & Johnson, Moderna, and CanSino. The country was the first in the region to begin vaccination and, in February 2021, ranked 7th in global vaccination ^50^.

Pfizer is present in almost all countries, except for Nicaragua, Venezuela, Guatemala, and Bolivia, which prioritized relations with Russia, China, and Cuba. Bolivia acquired Sputnik V (Russia), Sinopharm (China), and Johnson & Johnson vaccines. In addition to Pfizer, many countries bought vaccines from AstraZeneca and Sinovac.

The figures show different national vaccine supply strategies during the pandemic, considering access, international negotiations, and confidence in available vaccines. Diversification was common, with some countries buying vaccines from different manufacturers, while others preferred a more concentrated approach.

**Figure 3:**
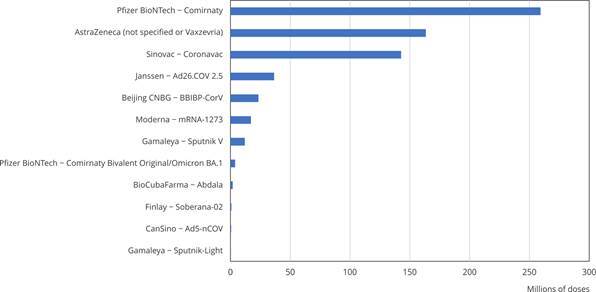
Total doses administered per manufacturer in Latin America and the Caribbean, December 2023.

Source: prepared by the authors based on Pan American Health Organization ^26^

Figure 3 shows the total number of vaccine doses administered in Latin America and the Caribbean, according to different producers. Comirnaty (Pfizer) was the most common vaccine in the region, exceeding 250 million doses, followed by AstraZeneca with about 163 million doses and Sinovac with 142 million doses.

Next are Janssen (Johnson & Johnson), BBIBP-CorV (Sinopharm), mRNA-1273 (Moderna), and Sputnik V (Gamaleya Institute), with 36, 23, 17, and 12 million doses administered, respectively. The Cuban vaccines Abdala (BioCubaFarma, Cuba) and Soberana-02 (Finlay Vaccine Institute, Cuba) appear in smaller quantities, used in Cuba, Venezuela, and Nicaragua.

## Discussion

### Determinants and challenges of inequalities in access to vaccines

The COVID-19 pandemic surprised even countries considered well-prepared to respond to emergencies. In Latin America and the Caribbean, structural inequalities exacerbated regional vulnerability in terms of cases, deaths, and vaccination coverage ^51^. The biggest challenge in the region was the scarcity of vaccines, due to its dependence on imports of pharmaceutical products and raw materials and the concentration of vaccine acquisition by developed countries in a scenario of limited supply.

The strategies adopted by WHO and PAHO to promote access to vaccines in the region were important, but they encountered structural, institutional, and conjunctural limitations. The structural limitations included inequalities between and within countries, insufficient international solidarity, restricted access to resources, and structural problems in health systems. The COVID-19 pandemic highlighted the discrepancy in R&D and vaccine production capacities worldwide, the vulnerability of global supply chains, and the region’s dependence on imports ^52^. Latin America and the Caribbean, which import ten times more pharmaceutical products than they export, are vulnerable to global variations in supply and demand during health emergencies ^5^.

A broad global movement of solidarity and search for solutions to address the pandemic was observed. However, these efforts must be accompanied by commitments and strategies to promote changes in international and national regulatory frameworks. In addition, voluntary initiatives are not universal and they do not reach all populations. The conflict between health and trade is evident, even during health crises ^53^.

Institutional limitations range from pre-existing weaknesses in multilateral organizations ^54^ and the capacities of national health systems to deal with the crisis, to challenges in transnational governance arrangements and mechanisms adopted in the pandemic response. Zamudio-González ^55^ identifies an indirect governance through orchestration, in which WHO and PAHO acted as “governors”, performing coordination roles with other agents that assumed crucial responsibilities voluntarily, without hierarchical or contractual ties. This type of arrangement is consistent with the administration of these organizations and can offer advantages in times of crisis. On the other hand, it depends on the power and legitimacy of these organizations, as well as the adherence of other actors, considering the pressures and uncertainties involved.

One example of governance through orchestration would be the COVAX mechanism, in which Gavi and CEPI had leadership roles in articulation with the WHO and UNICEF. This arrangement was defended by one of its creators for its decision and operational flexibility, which would not be possible within WHO’s organizational structure ^56^. Elder & Malter ^57^ argue that equity goals in access to vaccines were not achieved due to delays in vaccine delivery in low- and middle-income countries as a result of global competition and suggest that a government-led mechanism would be more legitimate to lead COVAX than a public-private partnership.

Another example is the decision about the use of the PAHO Revolving Fund integrated into COVAX, in which WHO sought to coordinate the globally established strategy − together with Gavi, CEPI, and UNICEF − with a vaccine distribution mechanism that previously existed only in the Americas, under the coordination of PAHO, a regional WHO agency that is unique for its long history ^16^ and relative autonomy.

The conjunctural problems, associated with the others described above, were related to decisions made during the emergency, given the imbalance between supply and demand for vaccines. Berkley ^56^ pointed out that “vaccine nationalism” and export ban would have harmed the results of COVAX. To ensure vaccination, wealthy nations and countries with purchasing power, such as Peru, Chile, and Brazil, acquired doses beyond what was needed to immunize their populations. This strategy limited COVAX deliveries, impacting its ability to promote equitable access to vaccines in the region.

Globally, there were disputes between developed countries, which prioritized their own needs or the particular interests of their leaders, highlighting the contrast between the notion of global public goods and the competitive logic of the market ^58^. The debate on public or common goods in health − which are not limited to the product, but include immunization ^59^ − was present internationally before the pandemic. Given the high volume of public resources invested in health innovation markets, the licensing of these products could be mandatory or, at least, subject to more stringent conditions to serve the public interest ^60^.

In this sense, recognizing products related to COVID-19 as global public goods would be a decisive step towards reducing barriers to access. For this reason, global governance that can regulate the functioning of markets, through transparency, fair pricing mechanisms. and the elimination of intellectual property barriers, is essential for an effective cooperation between States ^58^.

Faced with excess demand and government pressure, companies prioritized more advantageous market strategies, causing disruptions in vaccine supply during the pandemic ^3^. The interruption of exports in 2021 by the Serum Institute of India, one of the largest vaccine manufacturers in the world, which produced 50 million doses of the AstraZeneca vaccine ^61^ per month, affected agreements with AstraZeneca and the supply of Covax, which had bought 200 million doses from the Institute and almost 170 million doses from AstraZeneca ^62^ ^,^ ^63^.

Consequently, COVAX failed to meet its commitments at the global and regional levels. By September 2021, COVAX had distributed 190 million doses worldwide, representing 4% of the doses administered. By the end of 2021, less than 1 billion doses had been distributed, half of the target of 2 billion ^56^. Then, countries that depended on COVAX AMC were far from achieving their vaccination coverage goals ^3^. When COVAX ended in 2023, all 1.997 billion doses distributed had contributed significantly to vaccination in low-income countries, but inequalities in vaccination coverage were notable when compared to high-income countries ^56^.

In Latin America and the Caribbean, the fact that self-financed countries received only a portion of the doses bought by COVAX may have affected PAHO’s relationship with its Member States. The acquisition of vaccines with PAHO’s Revolving Fund was relevant, but the Fund could have been used earlier for other purposes − price negotiation, expansion of the vaccine portfolio, and financing mechanisms − which would have contributed to a more effective promotion of equitable access in the region.

The variation in the quantities of vaccines bought reveals differences in populations, national distribution capacities, and vaccination strategies. Although the COVAX initiative distributed a significant number of doses, it was not able to fulfill regional needs and help achieve the vaccination coverage goals.

The pandemic highlighted the need for national development strategies that guarantee independence in the production and innovation system ^64^. Technology transfer for vaccine production fell short of expectations for a region with important pharmaceutical sectors in different countries. Insufficient local production capacity left countries dependent on imports of vaccines or active pharmaceutical ingredients, aggravating the region’s vulnerability.

According to Lima & Gadelha ^19^, strengthening the Health Economic-Industrial Complex (HEIC) is very important, considering the bases of production, science, technology, and innovation as essential for the sustainability of health systems and global health. The authors highlight inequality in knowledge, innovation, and production in health as a challenge to be faced in order to reduce the dependence of countries on imports of strategic inputs, especially in the Global South, and achieve sustainable development with equity.

In addition to readjusting national strategies to strengthen science and local production and regional integration, the COVID-19 pandemic highlighted the need to update mechanisms for coordination and global solidarity. After more than three years of negotiation, the WHO Pandemic Agreement was approved, which addresses various topics, including access to vaccines. This agreement represents an important step forward, despite the need for regulation of controversial aspects ^54^.

Study limitations include gaps in data. Around 714 million doses administered in the region were registered without a manufacturer identification, indicating problems in registration made by countries and distorting analyses of vaccine coverage and effectiveness. There are differences in data due to the heterogeneity of national health information systems. Also, mortality data from Our World in Data were (https://ourworldindata.org/) not standardized by demographic structure of the countries. The interviews were conducted with a small number of key informants, facilitating the understanding of strategic decisions, challenges addressed, and lessons learned, complementing the document analysis and the secondary data analysis. Studies should be conducted to further analyze the agenda and decision-making processes of WHO and PAHO, in their relationship with other global health actors, such as companies operating in the sector and nation states. 

## Conclusions

Unequal access to vaccines and vaccination against COVID-19 and insufficient collaboration among Latin American and Caribbean countries reinforce the importance of strengthening regional cooperation mechanisms and international coordination by multilateral organizations. Despite the challenges, WHO’s actions were fundamental, and PAHO played a relevant role in coordinating the acquisition and distribution of vaccines in the region and supporting Member States in the introduction of vaccines and the continuity of vaccination in immunization programs.

In addition to global and regional strategies to promote access to vaccines, some countries developed their own initiatives, according to their capacities. Inequalities between countries, the diversity of strategies, and autonomous national responses influenced the actions of international organizations. The prioritization by governments of bilateral negotiations with pharmaceutical companies increased global competition for vaccines, weakening international cooperation. However, the variety of vaccines allowed countries to address supply and distribution restrictions during the pandemic. In this context, the need to consolidate more stable institutional arrangements for regional coordination in future health crises is evident, which would reduce the fragmentation of responses and allow a better alignment between governments. Also, health must be placed at the center of social and economic systems.

The technological dependence of countries is a critical aspect for understanding the difficulties and challenges of the strategies adopted by WHO and PAHO. Such dependence is alarming with the crisis of multilateralism, which weakens the role of these organizations and compromises WHO’s ability to set the global health agenda with autonomy and authority. The concentration of innovation and production reinforces inequalities and limits the effectiveness of multilateral responses. Strengthening local and regional vaccine production through cooperation between countries, with the support of WHO and PAHO, can reduce the region’s dependence on imports, representing an important strategy to address future health emergencies.

## Data Availability

The sources of information used in the study are indicated in the body of the article.
