# Supplementary material for: The COVID-19 pandemic and access to vaccines in Latin America and the Caribbean: strategies adopted by the WHO and PAHO
Source: Cad Saude Publica. 2026 Aug 3;42(Suppl 1):e00016225. doi: 10.1590/0102-311XEN016225 (PMC13435628; doi:10.1590/0102-311XEN016225)
Supplement: Supplementary file 1 [file 1678-4464-csp-42-s1-EN016225-s-pt.pdf]

Material Suplementar

Apêndice 1

Foi realizada uma busca bibliográfica de textos provenientes dos campos da saúde pública e das relações internacionais, já que o tema é interdisciplinar e os campos de conhecimento se complementam. O levantamento bibliográfico pôde mapear as publicações já existentes sobre o tema do acesso às vacinas de COVID-19, identificar os principais atores e sustentar a discussão conceitual. A busca foi feita na base bibliográficas Portal BVS e foram utilizados os seguintes descritores em ciências da saúde (DeCS, https://decs.bvsalud.org/): ("Iniquidades em Saúde" OR "Acesso a vacina" OR "Organização Mundial da Saúde" OR OMS OR "Organização Pan-Americana da Saúde" OR OPAS OR UNICEF OR "Coalition for Epidemic Preparedness Innovations" OR CEPI OR GAVI OR "The Vaccine Alliance") AND ("Vacinas contra COVID-19" OR "Vacina contra o COVID19" OR "Vacina contra o COVID-19" OR "Vacina contra o COVID 19" OR "Vacina contra o SARS-CoV-2" OR "Vacina contra o SARS2"). A busca abrange as publicações até agosto de 2022, mês em que se realizou a pesquisa, e resultou na identificação de 51 artigos.

Além disso, também foi realizada busca nas bases bibliográficas Scopus e Web of Science, com os seguintes DeCS: ("Access to Essential Medicines and Health Technologies" OR "Health Inequities" OR "Health Equity" OR "Access vaccines") AND ("World Health Organization" OR WHO OR "Pan American Health Organization" OR PAHO OR UNICEF OR "Coalition for Epidemic Preparedness Innovations" OR CEPI OR GAVI OR "The Vaccine Alliance") AND ("COVID-19 Vaccines" OR "COVID19 Vaccines" OR "COVID 19 Vaccines"). Como resultado foram identificados 64 artigos na Scopus e 10 na Web of Science.

A partir da rápida revisão dos títulos e resumos dos artigos identificados, foram selecionados 34 para leitura.
