# Supplementary material for: The COVID-19 pandemic and access to vaccines in Latin America and the Caribbean: strategies adopted by the WHO and PAHO
Source: Cad Saude Publica. 2026 Aug 3;42(Suppl 1):e00016225. doi: 10.1590/0102-311XEN016225 (PMC13435628; doi:10.1590/0102-311XEN016225)
Supplement: Supplementary file 2 [file 1678-4464-csp-42-s1-EN016225-s.pdf]

Supplementary material

Appendix 1

A bibliographic search was conducted in texts from the fields of public health and international relations, given the interdisciplinary nature of the topic and because these fields of knowledge complement each other. This bibliographic search mapped existing publications on access to COVID-19 vaccines, identified key actors, and supported the conceptual discussion. The search was conducted in the VHL Portal database using the following Health Sciences Descriptors (DeCS, https://decs.bvsalud.org/): (“Iniquidades em Saúde” OR “Acesso a vacina” OR “Organização Mundial da Saúde” OR WHO OR “Organização Pan Americana da Saúde” OR PAHO OR UNICEF OR “Coalition for Epidemic Preparedness Innovations” OR CEPI OR GAVI OR “The Vaccine Alliance”) AND (“Vacina contra o COVID-19” OR “Vacinas contra o COVID-19” OR “Vacina contra o COVID 19” OR “Vacina contra o COVID-19” OR “Vacina contra o SARS-CoV-2” OR “Vacina contra o SARS2”). The search covers publications up to August 2022, when the search was conducted, and it found 51 articles.

In addition, a search was conducted on SCOPUS and Web of Science, using the following DeCS: (“Access to Essential Medicines and Health Technologies” OR “Health Inequities” OR “Health Equity” OR “Access vaccines”) AND (“World Health Organization” OR WHO OR “Pan American Health Organization” OR PAHO OR UNICEF OR “Coalition for Epidemic Preparedness Innovations” OR CEPI OR GAVI OR “The Vaccine Alliance”) AND (“COVID-19 Vaccines” OR “COVID19 Vaccines” OR “COVID 19 Vaccines”). The search found 64 articles on SCOPUS and 10 articles on Web of Science.

After a quick review of the titles and abstracts of the identified articles, 34 articles were selected for reading.
