# Supplementary material for: The COVID-19 pandemic and access to vaccines in Latin America and the Caribbean: strategies adopted by the WHO and PAHO
Source: Cad Saude Publica. 2026 Aug 3;42(Suppl 1):e00016225. doi: 10.1590/0102-311XEN016225 (PMC13435628; doi:10.1590/0102-311XEN016225)
Supplement: Translation

# Translation: Pandemia de COVID-19 e acesso a vacinas na América Latina e Caribe: estratégias da OMS e da OPAS

**DOI:** 10.1590/0102-311XPT016225

**Authors:** Luana Bermudez, Cristiani Vieira Machado

**Affiliations:** 1 Escola Nacional de Saúde Pública Sergio Arouca, Fundação Oswaldo Cruz, Fiocruz, Rio de Janeiro, Brasil.

**Keywords:** Pandemia de COVID-19, Acesso a Medicamentos Essenciais e Tecnologias em Saúde, Vacinas, América Latina, COVID-19 Pandemic, Access to Essential Medicines and Health Technologies, Vaccines, Latin America, Pandemia de COVID-19, Acceso a Medicamentos Esenciales y Tecnologías Sanitarias, Vacunas, América Latina

## Abstract

This study analyzes the strategies of the World Health Organization (WHO) and the Pan American Health Organization (PAHO) to promote access to COVID-19 vaccines in Latin America and the Caribbean, exploring the vaccines administered, vaccination coverage in the countries, and inequalities in vaccination in the region. The study method combines analysis of secondary data and of documents, and interviews with key informants. The results show advances and limitations of the WHO and PAHO initiatives to promote vaccine equity, adopt redistributive measures to reduce inequalities in access to vaccines, and achieve the established global coverage goals. This study identified structural and political-institutional factors that favored or hindered the action of these organizations in vaccine distribution and promotion of vaccination equity during the pandemic. The role of these multilateral organizations in global and regional health arrangements is critically discussed in terms of their legitimacy, institutional capacity, and technical competence. The conclusions highlight the importance of strengthening mechanisms of global solidarity and helping Latin American nations overcome structural problems, such as limited national production and scientific and technological dependence, which hampered vaccination processes and aggravated inequalities between countries during the health emergency.

## Introdução

A pandemia de COVID-19 agravou as desigualdades sociais na América Latina e Caribe ao atingir uma região já marcada por baixo crescimento econômico, investimentos insuficientes em políticas públicas e instabilidade política ^1^ ^,^ ^2^. O fraco desempenho econômico regional antes da crise, com crescimento médio anual de 0,3% entre 2014 e 2019 ^3^, deu lugar à retração econômica em 2020 com queda de 6,8% no produto interno bruto (PIB), a pior entre as regiões em desenvolvimento ^4^. Na segunda metade de 2021, a inflação alcançou 6,6%, um patamar não visto desde outubro de 2008 ^5^, enquanto o desemprego aumentou entre 2019 e 2022, aprofundando a crise. A taxa de mortalidade aumentou, a esperança de vida ao nascer diminuiu, e o PIB total e *per capita* recuaram entre 2020 e 2022, refletindo o impacto profundo sobre a dinâmica econômica e social regional.

Ademais, a região registrou excesso de mortalidade significativo, indicador que abrange mortes causadas pelo vírus e por outras causas no cenário da pandemia. Em 2020 e 2021, contabilizou-se um excesso de 2.273.620 mortes em relação ao previsto com base nas médias dos anos anteriores ^6^, correspondendo a 15% do excesso de mortes no mundo ^7^, apesar de a região abrigar 8% da população mundial. Em 2020, dos 10 países com o maior percentual de mortes em excesso no mundo, nove eram latino-americanos ^8^ ^,^ ^9^.

A Assembleia Geral das Nações Unidas de 2020 ressaltou a importância da solidariedade global para enfrentar a pandemia de COVID-19 e da cooperação internacional para assegurar o acesso a medicamentos, vacinas e equipamentos médicos ^10^ ^,^ ^11^, e a Assembleia Mundial da Saúde ^12^ aprovou resolução recomendando “*acesso universal, oportuno equitativo e justo a todos os produtos essenciais seguros, eficazes e acessíveis*, (...) *necessários na resposta à pandemia como prioridade global*” ^13^.

A Organização Mundial da Saúde (OMS), criada em 1948 para ser autoridade em saúde global ^14^ ^,^ ^15^, buscou assumir a liderança da resposta à COVID-19, contando com suas representações regionais. Entre elas, destaque-se a Organização Pan-Americana da Saúde (OPAS), criada em 1902, uma das mais antigas iniciativas de cooperação multilateral do mundo ^16^. A OPAS tem duas funções institucionais: escritório regional da OMS e agência especializada do Sistema Interamericano. Ela desempenha papel essencial na resposta regional a emergências sanitárias, mediante suporte técnico e coordenação de esforços entre países.

Tais agências enfrentam uma crise marcada pela perda de protagonismo, escassez de financiamento, fragmentação da governança e críticas à sua capacidade de resposta em emergências sanitárias. O surgimento de novos atores no campo da saúde trouxe competição e fragmentação, ampliando questionamentos sobre sua eficácia e credibilidade. Apesar do desgaste e de ataques, agravados durante a pandemia, a OMS continua sendo a única organização com legitimidade para liderar a saúde global ^17^ ^,^ ^18^, e a OPAS se mantém como agência de fundamental importância regional.

Em um cenário de assimetrias globais no desenvolvimento econômico, científico e tecnológico, diante de emergências sanitárias, as diferenças entre os que têm acesso a produtos e serviços de saúde e os que não têm acentuam inequidades entre povos e podem levar a crises humanitárias. A crescente concentração de capacidade produtiva e de inovação e a monopolização no setor saúde, sem mecanismos internacionais adequados de promoção da equidade e redistribuição entre os países, inviabilizam o alcance da universalidade em saúde ^19^.

Muitos determinantes das condições e das desigualdades em saúde ultrapassam o alcance dos governos nacionais e do setor saúde, exigindo soluções políticas articuladas em escala global ^20^. Contudo, a pandemia de COVID-19 demonstrou que a arquitetura da inovação não está estruturada para garantir acesso universal aos produtos biomédicos, pois o sistema de propriedade intelectual favorece concorrência sigilosa, monopólios e práticas que aprofundam a dependência de países de baixa renda ^21^. Os desafios para assegurar acesso equitativo aos frutos da pesquisa & desenvolvimento (P&D) biomédica reforçam, portanto, a urgência de reorientar esse sistema para o interesse público ^22^. Isso requer estratégias para fortalecer capacidades científicas e industriais em todas as regiões, e regras que assegurem o compartilhamento global de conhecimento e tecnologia. A expansão de polos regionais de produção é um passo essencial para enfrentar crises atuais e para preparar o mundo para futuras emergências sanitárias ^23^.

A OMS estabeleceu a meta de vacinar 70% da população mundial, com o objetivo de conter a pandemia com a maior celeridade possível. O desenvolvimento e o acesso oportuno e equitativo a vacinas seguras e eficazes seriam decisivos para reduzir casos graves e mortes e alcançar o fim da pandemia de COVID-19. Nesse cenário, a OPAS apoiou seus países-membros nas respostas à pandemia. Apesar das diferentes estratégias nacionais, todos implementaram medidas de vacinação.

Diante da relevância dessas agências no cenário internacional e da importância do acesso a insumos estratégicos e da vacinação para a saúde pública, é importante explorar como a OMS e a OPAS atuaram para garantir o acesso a vacinas para COVID-19 e reduzir desigualdades na vacinação entre países. Dado o caráter recente da pandemia, ainda há relativa escassez de estudos sobre o tema.

Este artigo objetiva analisar as estratégias empregadas pelas duas organizações para promover o acesso à vacinação contra COVID-19 e descrever as vacinas administradas e a cobertura vacinal nos países da América Latina e Caribe, considerando as desigualdades na vacinação.

## Método

Trata-se de um estudo exploratório descritivo com ênfase na atuação da OMS e da OPAS na promoção do acesso a vacinas de COVID-19, orientado por quatro eixos de análise:

a) Contexto socioeconômico e epidemiológico dos países da América Latina e Caribe de 2019 a 2023;

b) Estratégias de promoção do acesso às vacinas contra COVID-19 adotadas pela OMS e pela OPAS;

c) Cobertura vacinal e vacinas utilizadas na região;

d) Condicionantes e desafios das desigualdades no acesso às vacinas na região.

O estudo baseou-se em dados secundários e análise documental, complementados por entrevistas com informantes-chave.

O levantamento de dados secundários foi realizado em diferentes fontes: os anuários estatísticos da Comissão Econômica para a América Latina e o Caribe (CEPAL) de 2019 a 2023 ^24^ para dados socioeconômicos; a plataforma Our World in Data ^25^ para dados epidemiológicos; a base de dados da OMS sobre excesso de mortalidade associada à COVID-19 ^7^, o repositório do Financial Times para excesso de mortalidade de 2020 a 2021 ^18^, os *dashboards* da OPAS sobre vacinação e entregas da COVAX ^26^ ^,^ ^27^.

Analisaram-se documentos oficiais, relatórios e comunicados disponíveis nos sites da OMS (https://www.who.int/), da OPAS (https://www.paho.org/en) e da CEPAL (https://www.cepal.org/), relativos à pandemia, iniciativas de cooperação internacional e estratégias regionais de promoção do acesso às vacinas de COVID-19. O período analisado foi de 2020 a 2023, abrangendo do início da pandemia até a consolidação das estratégias de vacinação. Consideraram-se diferentes fontes, como notas técnicas, relatórios, comunicados de imprensa e planos relacionados à cooperação internacional e ao acesso às vacinas contra COVID-19.

Complementarmente, realizaram-se seis entrevistas semiestruturadas com informantes-chave sobre as estratégias da OMS e da OPAS. Os critérios de seleção dos entrevistados foram: especialista em vacinas, participação nas iniciativas, nos conselhos ou grupos de especialistas, como a Coalizão para Inovações em Preparação para Epidemias (CEPI, *Coalition for Epidemic Preparedness Inovation*), GAVI, a Aliança da Vacina (Gavi, *The Vaccine Alliance*) e o Grupo Técnico Consultivo (TAG) do Grupo de Acesso às Tecnologias contra a COVID-19 (C-TAP, *COVID-19 Technology Access Pool*).

Os seis entrevistados (3 da OMS, 2 da OPAS e 1 CEPI) foram convidados a participar da pesquisa por e-mail, com a explicitação dos objetivos. As entrevistas foram realizadas presencialmente (1) e on-line (5), conforme a disponibilidade do entrevistado. Cada entrevista durou cerca de uma hora e todas foram gravadas e transcritas pela pesquisadora. Procedeu-se à análise de conteúdo segundo os eixos do estudo, sem uso de softwares. Na apresentação dos resultados, garantiu-se confidencialidade utilizando a letra “E” seguida de um número.

Adicionalmente, realizou-se pesquisa bibliográfica para mapear publicações sobre o acesso a vacinas contra a COVID-19, de 2020 a 2022. As buscas foram realizadas no Portal BVS, Scopus e Web of Science com Descritores em Ciências da Saúde (DeCS) relacionados ao tema (Material Suplementar: Apêndice 1; https://cadernos.ensp.fiocruz.br/static//arquivo/suppl-e00016225-port_8574.pdf), com recuperação respectivamente de 51, 64 e 10 artigos, dos quais foram selecionados 34 para leitura.

O projeto de pesquisa foi aprovado por Comitê de Ética em Pesquisa da Escola Nacional de Saúde Pública Sergio Arouca, Fundação Oswaldo Cruz (CAAE 60435522.6.0000.5240). 

## Resultados

### Estratégias de acesso a vacinas da OMS e da OPAS

A principal estratégia da OMS foi o Acelerador de Acesso às Tecnologias contra a COVID-19 (ACT-A, *Access to COVID-19 Tools Accelerator*), consórcio global lançado em 2020, envolvendo mais de 40 países para o desenvolvimento, produção e acesso a diagnósticos, terapêuticas e vacinas contra COVID-19 ^28^.

No âmbito do ACT-A, lançou-se o mecanismo COVAX (*COVID-19 Vaccines Global Access*) para acelerar o desenvolvimento, aquisição e distribuição de vacinas, coordenado pela CEPI, Gavi e OMS, em colaboração com Fundo das Nações Unidas para a Infância (UNICEF). A CEPI liderou atividades de P&D; a Gavi, a compra e distribuição em larga escala; a OMS, a orientação sobre políticas de vacinas, regulamentação, segurança, P&D, alocação, preparação e entrega nos países. O UNICEF apoiou na aquisição de vacinas, transporte e logística ^29^.

O objetivo do COVAX era disponibilizar 2 bilhões de doses de vacinas até dezembro de 2021, calculado com base no número mundial de pessoas de alto risco e profissionais de saúde na linha de frente ^30^. O mecanismo compreendeu duas formas de participação: países poderiam participar do COVAX *Advance Market Commitment* (AMC, Compromisso Antecipado de Mercado), financiado por doações; ou autofinanciar a compra de doses para vacinar entre 10% e 50% de suas populações ^30^. Até agosto de 2020, 172 países, representando mais de 70% da população mundial, aderiram ao mecanismo, 80 autofinanciados e 92 pelo COVAX AMC ^31^.

Outra estratégia da OMS foi o C-TAP, lançado em 2020 em parceria com a Costa Rica ^32^. O C-TAP visava promover acordos de compartilhamento de propriedade intelectual e know-how pelos fabricantes de produtos, para facilitar o aumento da produção e reduzir barreiras de acesso ^33^.

Apesar de a OMS defender a distribuição equitativa pelo COVAX e o compartilhamento de tecnologias pelo C-TAP, ficou evidente, no início de 2021, que os países em desenvolvimento teriam dificuldades para receber vacinas contra a COVID-19. Em 2021, a OMS convocou empresas interessadas em sediar um centro de transferência de tecnologia de RNA mensageiro (mRNA) e, em junho desse ano, selecionou um consórcio sul-africano. A agência criou o Programa de Transferência de Tecnologia de mRNA, visando desenvolver capacidades sustentáveis de produção em países em desenvolvimento ^34^. Embora crucial para o acesso a tecnologias, essa iniciativa não atendeu à fase aguda da pandemia, cuja necessidade imediata era a distribuição rápida e equitativa das vacinas disponíveis. O Programa seria uma solução a longo prazo que visa fortalecer a capacidade de produção local para prevenção e resposta a futuras emergências sanitárias.

Nas Américas, a OPAS foi fundamental na implementação de iniciativas de acesso a vacinas durante a pandemia. Entre elas, incentivou a doação de vacinas para a região, negociando com governos o compartilhamento de doses, e apoiou a operacionalização do COVAX, inclusive na redistribuição de doses entre países ^35^.

O objetivo do COVAX na região era fornecer vacinas para imunizar 20% da população de cada país participante até o fim de 2021 ^36^. Como o COVAX não alcançou o sucesso esperado no primeiro ano, em agosto de 2021 a OPAS decidiu utilizar o Fundo Rotatório para adquirir vacinas e complementar o fornecimento para os Estados-membros ^37^. Este Fundo, criado em 1979, é um mecanismo de aquisição de vacinas, representando um bloco de 42 países, que negocia reduções de preços para garantir a sustentabilidade dos programas nacionais de imunização ^38^.

Além disso, a OPAS lançou uma convocatória para empresas regionais participarem do Programa de Transferência de Tecnologia de mRNA da OMS ^39^. Em 2021, anunciou-se que Bio-Manguinhos/FIOCRUZ (Instituto de Tecnologia em Imunobiológicos, Fundação Oswaldo Cruz, Brasil) e Sinergium Biotech (Argentina) foram selecionados, para fortalecer sua capacidade produtiva e reduzir a dependência de importações da região diante de emergências sanitárias ^40^.

No mesmo ano, a Organização propôs a Plataforma Regional para o Avanço na Produção de Vacinas e Outras Tecnologias em Saúde para COVID-19 nas Américas, visando reunir parceiros públicos e privados para impulsionar P&D e a produção de tecnologias, e promover colaboração entre países e agências para utilização da capacidade de biomanufatura existente ^41^. A seleção da OPAS de produtores de mRNA e a criação da Plataforma Regional têm potencial de impacto em crises sanitárias futuras, ao garantir infraestrutura regional para a produção e distribuição de vacinas e tecnologias em saúde, reduzindo a dependência de importações e promovendo a equidade no acesso a insumos de saúde.

Ademais, a OPAS apoiou os países no recebimento das vacinas, mediante planejamento da demanda, logística e gestão da cadeia de frio, fortalecimento da vigilância de eventos adversos e dos sistemas de informação, treinamento de profissionais de saúde, geração de demanda por vacinas e diretrizes para comunicação de risco.

As estratégias adotadas pelas duas organizações visaram promover a equidade no acesso às vacinas contra COVID-19, embora sob desafios, devido à competição global e limitações de produção, que foram abordados pelos entrevistados.

Segundo um entrevistado (E1), diversos fatores desequilibraram a alocação por meio do COVAX, como a interrupção do envio de vacinas pela Índia, quando a pandemia atingiu duramente o país, e a atuação de um consórcio europeu para a compra conjunta de vacinas. O bloco processou a AstraZeneca por quebra de contrato e tentou forçar a entrega de 20 milhões de doses da Índia para o consórcio. Muitas farmacêuticas não cumpriram prazos de entrega previstos nos contratos.

Tais dificuldades foram ressaltadas por outro entrevistado:

“*O maior desafio operacional foi na primavera de 2021, quando não conseguimos acessar vacinas da Índia. Países de alta renda compraram todas as vacinas, e o Serum Institute não podia exportar. Gavi não tinha acesso imediato a fundos para fazer acordos de compra antecipada necessários para estar na frente da fila*” (E6, nossa tradução).

Alguns entrevistados enfocaram desafios enfrentados na relação entre OPAS, OMS e outras organizações. O entrevistado E1 mencionou que a inclusão do Fundo Rotatório no COVAX enfrentou resistências. No entanto, como outras regiões não possuíam fundo equivalente, foi possível incluí-lo como mecanismo de compras para as Américas.

O entrevistado E4 mencionou que a decisão de incluir o Fundo Rotatório para complementar a aquisição de vacinas do COVAX foi tomada no alto comando da OMS, diante dos atrasos e da tendência dos países latino-americanos ficarem no final da fila para receber vacinas. O Fundo poderia ter sido utilizado desde o início da pandemia para garantir uma cobertura vacinal equitativa ^42^ porque, ao projetar maior escala de demanda, reduz o preço das vacinas e amplia o poder de compra dos Estados ^38^. Um entrevistado abordou o papel da OPAS na governança do COVAX:

“*Éramos um dos dois agentes de aquisição*. (...) *papel importante, mas deveríamos ter tido papel maior. Participamos de chamadas do COVAX, mas não no nível mais alto. Éramos convidados para referências específicas a ações de aquisição. Não participamos das discussões do AMC com fornecedores*. (...) *O UNICEF tinha mais acesso; mantínhamos nossos canais com a OMS*. (...) *A equipe estava envolvida em componentes da discussão, mas não tínhamos autoridade para tomar decisões*” (E4, nossa tradução).

O entrevistado ressaltou que os diretores regionais participavam de chamadas semanais de atualização à OMS. Em sua opinião, a atenção da OMS à América Latina e ao Caribe era menor do que a dedicada a outras regiões, especialmente à África. Assim, considera que a OPAS poderia ter feito mais:

“*A liderança na época estava focada em fazer o que fizemos, até o verão de 2021, quando abrimos o terceiro componente da nossa estratégia regional. Isso foi tarde demais*” (E4, nossa tradução).

Segundo a OPAS ^42^, dois fatores afetaram sua capacidade de resposta: a centralização das decisões na OMS e a dependência do COVAX para adquirir e distribuir vacinas. Destacam-se fatores internos, como crise financeira e de recursos humanos, além de dificuldades de comunicação entre a sede da OPAS e os escritórios nos países.

Outro problema é que os países autofinanciados podiam comprar doses para vacinar até 50% de suas populações, enquanto os países do COVAX AMC só podiam receber doses para 20% de sua população, o que exacerbou desigualdades ^42^. Acrescente-se a variabilidade nos preços praticados: a vacina Moderna nos Estados Unidos custou USD 15, enquanto na Argentina custou 50% mais ^3^.

Apesar das dificuldades, E1 defendeu a integração do Fundo Rotatório ao COVAX, pois a compra direta pela OPAS exacerbaria a competição global e a escassez de vacinas. O entrevistado argumentou que o COVAX ajudou países que não teriam acesso às vacinas.

Outro entrevistado (E5) destacou que a Plataforma Regional foi útil para promover a interação entre produtores locais, reforçar a produção e as capacidades regulatórias regionais, com expectativa de impacto em futuras crises.

Ressalte-se que, além das iniciativas voltadas à distribuição de vacinas, a OMS e a OPAS realizaram outras ações para fortalecer a vacinação nos países, como capacitação e organização das redes de cadeia fria.

### Vacinação na América Latina e Caribe

Países latino-americanos e caribenhos tiveram acesso às vacinas por meio do COVAX, de doações e de negociações bilaterais para a compra ou a transferência de tecnologia. A maioria aderiu ao COVAX: 23 países participaram, 13 se autofinanciaram e dez aderiram ao AMC ^42^.

A vacinação na região começou em dezembro de 2020 no Chile, México, Costa Rica e Argentina, e em janeiro de 2021 no Brasil ^43^. A partir de março de 2021, países latino-americanos receberam vacinas do COVAX, em quantidades reduzidas ^44^, que somaram 77,8 milhões de doses em 2021 ^45^ e 155 milhões até outubro de 2022, representando 10% do total de vacinas na região. Dessas doses, 73% foram viabilizadas pelo Fundo Rotatório, enquanto 27% foram adquiridas pelos países. Além disso, 32% das vacinas foram doadas por países ou financiadas pelo COVAX AMC, enquanto 68% foram autofinanciadas. Os países da região receberam cerca de 30% das doses contratadas pelo COVAX ^42^.

As sub-regiões mais beneficiadas por doações internacionais foram o Caribe, a América Central e a zona andina, que receberam doses de países desenvolvidos ^5^.

A Figura 1 mostra assimetrias na vacinação. Chile, Cuba e Nicarágua apresentaram as maiores coberturas vacinais, enquanto Haiti, Jamaica e Santa Lúcia, as menores. Em dezembro de 2022, na maioria dos países latino-americanos, mais de 70% da população estava vacinada. No Caribe, quatro países alcançaram 70% da população vacinada, e sete tinham menos de 40%, meta estabelecida pela OMS para o fim de 2021.

**Figura 1:** Cobertura vacinal (% da população) por país e média da região da América Latina e Caribe, de dezembro de 2020 a dezembro de 2022.

Fonte: elaboração própria, a partir de dados do site Our World in Data (Mathieu et al. ^25^). Nota: percentual da população com esquema vacinal inicial completo de duas doses ou dose única.

As estratégias nacionais variaram. Países mais populosos destacaram-se entre os que contrataram mais de 100 milhões de doses (Figura 2a), exceto a Colômbia. O Brasil priorizou a produção local, apostando inicialmente em acordos de compra antecipada e transferência de tecnologia com AstraZeneca (Inglaterra) e Sinovac Biotech (China) ^46^ ^,^ ^47^ ^,^ ^48^. Posteriormente, contratou doses da Pfizer (Estados Unidos), Johnson & Johnson (Canadá), CanSino (China) e Moderna (Estados Unidos), refletindo diversificação de fornecedores, enquanto fortalecia sua capacidade produtiva nacional. Já Argentina e México apostaram na diversificação de contratação e também fizeram acordo com a AstraZeneca para transferência de tecnologia ^49^, mas tiveram dificuldades na produção. O Peru contratou o equivalente a cinco doses por pessoa, de diversos fornecedores, com predominância da Pfizer.

**Figura 2:** Vacinas contratadas por países da América Latina, por produtor.

Fonte: elaboração própria a partir de dados de Harrison et al. ^43^. Nota: os dados foram separados em dois devido à escala dos gráficos.

Na Figura 2b, sobressai o Chile, com 20 milhões de habitantes, que contratou mais de 80 milhões de doses, com destaque para Sinovac, seguida pela Pfizer, AstraZeneca, Johnson & Johnson, Moderna e CanSino. O país foi o primeiro da região a iniciar a vacinação e, até fevereiro de 2021, ocupava o 7º lugar no ranking mundial de vacinação ^50^.

Observa-se a presença da Pfizer em quase todos os países, exceto na Nicarágua, Venezuela, Guatemala e Bolívia, que priorizaram as relações com Rússia, China e Cuba. A Bolívia adquiriu vacinas Sputnik V, Sinopharm e Johnson & Johnson. Além da Pfizer, muitos países contrataram doses da AstraZeneca e Sinovac Biotech.

As figuras mostram diferentes estratégias nacionais de fornecimento de vacinas durante a pandemia, considerando acesso, negociações internacionais e confiança nas vacinas disponíveis. A diversificação foi comum, alguns países contrataram vacinas de diferentes fabricantes, enquanto outros preferiram uma abordagem mais concentrada.

A Figura 3 mostra o total de doses de vacinas administradas na América Latina e Caribe, segundo diferentes produtores. Comirnaty (Pfizer) foi a vacina mais administrada na região, ultrapassando 250 milhões de doses, seguida pela AstraZeneca com cerca de 163 milhões de doses e Sinovac, com 142 milhões.

**Figura 3:** Total de doses administradas por produtor na América Latina e Caribe, dezembro 2023.

Fonte: elaboração própria a partir de dados de Organização Pan-Americana da Saúde ^26^.

A seguir, estão a Janssen (Johnson & Johnson), BBIBP-CorV (Sinopharm, China), mRNA-1273 (Moderna) e Sputnik V (Gamaleya, Russia), com, respectivamente, 36, 23, 17 e 12 milhões de doses administradas. As vacinas cubanas Abdala (BioCubaFarma, Cuba) e Soberana-02 (Instituto Finlay de Vacina, Cuba) aparecem em quantidades menores, utilizadas em Cuba, Venezuela e Nicarágua.

## Discussão

### Condicionantes e desafios das desigualdades no acesso às vacinas

A pandemia de COVID-19 surpreendeu inclusive países considerados bem preparados para responder a emergências. Na América Latina e Caribe, as desigualdades estruturais agravaram a vulnerabilidade regional, em termos de casos, mortes e cobertura vacinal ^51^. A maior dificuldade da região foi a escassez de vacinas, devido à dependência de importações de produtos farmacêuticos e de matérias-primas, mas também à concentração de compras pelos países desenvolvidos, em cenário de oferta limitada.

As estratégias adotadas pela OMS e pela OPAS para promover o acesso às vacinas na região foram importantes, mas esbarraram em limitações estruturais, institucionais e conjunturais. Entre as limitações estruturais, destacam-se as desigualdades entre e intra países, a insuficiente solidariedade internacional, o acesso restrito a recursos e problemas estruturais dos sistemas de saúde. A pandemia de COVID-19 evidenciou a disparidade nas capacidades de P&D e produção de vacinas no mundo, a vulnerabilidade das cadeias globais de suprimento e a dependência da região de importações ^52^. A América Latina e o Caribe, que importam dez vezes mais produtos farmacêuticos do que exportam, tornam-se vulneráveis a variações globais na oferta e demanda durante emergências ^5^.

Observou-se um amplo movimento global de solidariedade e busca por soluções para o enfrentamento da pandemia. Contudo, tais esforços devem ser acompanhados de compromissos e estratégias de mudança nos marcos regulatórios internacionais e nacionais. Além disso, iniciativas voluntárias não são universais nem alcançam todas as populações. O embate entre saúde e comércio é evidente, mesmo durante crises sanitárias ^53^.

Os limites institucionais abarcam desde fragilidades prévias das agências multilaterais ^54^ e das capacidades dos sistemas nacionais de saúde para lidar com a crise, até desafios dos arranjos de governança transnacional e mecanismos adotados na resposta à pandemia. Zamudio-González ^55^ identifica que se configurou uma governança indireta por orquestração, em que OMS e OPAS atuaram como “governadoras”, desempenhando funções de coordenação junto a outros agentes que assumiram responsabilidades cruciais, de forma voluntária, sem vinculação hierárquica ou contratual. Esse tipo de arranjo é coerente com os mandatos dessas agências e pode ter vantagens em momentos de crise. Por outro lado, depende do poder e legitimidade dessas organizações, bem como da adesão dos demais atores, em meio a pressões e incertezas de várias ordens.

Um exemplo de governança por concertação seria o arranjo do COVAX, em que Gavi e CEPI exerceram funções de liderança, em articulação com a OMS e UNICEF. Tal arranjo foi defendido por um de seus formuladores por sua flexibilidade decisória e operacional, que não seria possível dentro da estrutura organizacional da OMS ^56^. Já Elder & Malter ^57^ destacam que as metas de equidade no acesso não foram alcançadas diante de atrasos no recebimento de vacinas em países de baixa e média renda diante da competição global e sugerem que um mecanismo liderado pelos governos seria mais legítimo para liderar o COVAX do que uma parceria público-privada.

Outro exemplo seria a própria decisão relativa à utilização do Fundo Rotatório da OPAS, de forma integrada ao COVAX, em que a OMS buscou concertar a estratégia estabelecida mundialmente - junto a Gavi, CEPI e UNICEF - com um mecanismo de distribuição de vacinas previamente existente somente nas Américas, sob coordenação da OPAS, agência regional da OMS singular por sua longa trajetória histórica ^16^ e relativa autonomia.

Os problemas conjunturais, associados aos demais, relacionaram-se a decisões tomadas durante a emergência, diante do desequilíbrio entre oferta e demanda por vacinas. Berkley ^56^ apontou que o “nacionalismo vacinal” e vetos às exportações teriam prejudicado os resultados do COVAX. Para garantir a vacinação, nações ricas e países com capacidade de compra, como Peru, Chile e Brasil, adquiriram doses acima do necessário para imunizar suas populações. Essa estratégia limitou as entregas do COVAX, impactando sua capacidade de promoção de acesso equitativo às vacinas na região.

Mundialmente, houve disputas entre países desenvolvidos, que priorizaram suas próprias necessidades ou interesses particulares de seus governantes, evidenciando o contraste entre a noção de bem público global e a lógica competitiva do mercado ^58^. O debate sobre bens públicos ou comuns em saúde - que não se restringem ao produto, mas incluem a imunização ^59^ - estava presente internacionalmente antes da pandemia. Diante do elevado volume de recursos públicos investidos nos mercados de inovação em saúde, o licenciamento destes produtos poderia ser obrigatório ou, no mínimo, sujeito a condições mais rigorosas para atender ao interesse público ^60^.

Nesse sentido, reconhecer os produtos relacionados à COVID-19 como bens públicos globais seria um passo decisivo para reduzir barreiras de acesso. Para isso, uma governança global capaz de regular o funcionamento dos mercados, por meio de transparência, mecanismos justos de precificação e eliminação de barreiras de propriedade intelectual, é fundamental para que a cooperação entre os Estados seja efetiva ^58^.

Diante do excesso de demanda e das pressões dos governos, as empresas priorizaram estratégias de mercado mais vantajosas, com interrupções no fornecimento durante a pandemia ^3^. A interrupção de exportações, em 2021, do Instituto Serum da Índia, um dos maiores fabricantes mundiais, que produzia 50 milhões de doses/mês da vacina AstraZeneca ^61^, impactou acordos com a AstraZeneca e o suprimento do COVAX, que havia contratado 200 milhões de doses do Instituto e quase 170 milhões de doses da AstraZeneca ^62^ ^,^ ^63^.

Consequentemente, o COVAX não cumpriu os compromissos em nível global e regional. Até setembro de 2021, o COVAX havia distribuído 190 milhões de doses no mundo, o que representava 4% das doses administradas. Ao final de 2021, haviam sido distribuídas menos de 1 bilhão de doses, metade da meta de 2 bilhões ^56^. Assim, países dependentes do COVAX AMC estavam longe das metas de cobertura vacinal ^3^. Quando o COVAX foi encerrado em 2023, o número de 1,997 bilhão de doses distribuídas havia contribuído muito para a vacinação nos países de baixa renda, mas as desigualdades na cobertura vacinal em relação aos de renda alta eram marcantes ^56^.

Na América Latina e Caribe, o fato de os países autofinanciados terem recebido apenas parte das doses contratadas pelo COVAX pode ter afetado a relação da OPAS com seus Estados-membros. A aquisição de vacinas pelo Fundo Rotatório da OPAS foi relevante, mas o Fundo poderia ter sido utilizado antes, com outras atribuições - negociação de preços, expansão do portfólio de vacinas e de mecanismos de financiamento -, o que contribuiria para uma resposta mais efetiva na promoção do acesso equitativo na região.

A variação nas quantidades de vacinas contratadas reflete diferenças nas populações, nas capacidades nacionais de distribuição e nas estratégias de vacinação. Embora a iniciativa COVAX tenha distribuído uma quantidade significativa de doses, ficou aquém das necessidades regionais para atingir a meta de cobertura vacinal.

A pandemia evidenciou a necessidade de estratégias nacionais de desenvolvimento que garantam a independência no sistema produtivo e de inovação ^64^. A transferência de tecnologia para a produção de vacinas foi aquém do esperado para uma região com importantes setores farmacêuticos em diferentes países. A insuficiente capacidade de produção local deixou os países dependentes de importações de vacinas ou de ingredientes farmacêuticos ativos, o que exacerbou a vulnerabilidade da região.

Segundo Lima & Gadelha ^19^, é fundamental o fortalecimento do Complexo Econômico-Industrial da Saúde (CEIS), considerando as bases de produção, ciência, tecnologia e inovação como essenciais para a sustentabilidade dos sistemas de saúde e da saúde global. Os autores destacam a desigualdade em conhecimento, inovação e produção em saúde como um desafio a enfrentar para reduzir a dependência dos países de importações de insumos estratégicos, especialmente no Sul Global, e alcançar o desenvolvimento sustentável com equidade.

Além da reorientação das estratégias nacionais de fortalecimento da ciência e da produção local e de integração regional, a pandemia de COVID-19 evidenciou a necessidade de atualização dos mecanismos de coordenação e de solidariedade global. Após mais de três anos de negociação, foi aprovado o Acordo sobre Pandemias, que aborda diversos temas, inclusive o acesso a vacinas. Tal acordo representa um avanço importante, apesar da necessidade de regulamentação de pontos polêmicos ^54^.

Entre as limitações do estudo, destacam-se lacunas nos dados. Cerca de 714 milhões de doses administradas na região foram registradas sem identificação do produtor, o que indica problemas no registro pelos países e distorce análises sobre cobertura e efetividade vacinal. Há diferenças na confiabilidade dos dados devido à heterogeneidade dos sistemas nacionais de informação em saúde. Ademais, os dados de mortalidade da fonte Our World in Data (https://ourworldindata.org/) não foram padronizados por estrutura demográfica dos países. As entrevistas foram realizadas com número reduzido de informantes-chave, mas favoreceram a compreensão das decisões estratégicas, desafios enfrentados e lições aprendidas, em complementação à análise documental e de dados secundários. Sugere-se a realização de estudos que aprofundem a análise da agenda e processos decisórios na OMS e na OPAS, em sua relação com outros atores da saúde global, como empresas atuantes no setor e Estados nacionais.

## Conclusões

As desigualdades no acesso a vacinas e na vacinação contra COVID-19 e a insuficiente colaboração entre os países latino-americanos e caribenhos reiteram a relevância de fortalecer mecanismos de cooperação regional e coordenação internacional pelos organismos multilaterais. Apesar das dificuldades, a atuação da OMS foi fundamental e a OPAS desempenhou um papel relevante ao coordenar a aquisição e distribuição de vacinas na região, bem como no apoio aos Estados-membros na introdução das vacinas e continuidade da vacinação nos programas de imunização.

Além das estratégias globais e regionais de promoção do acesso a vacinas, alguns países desenvolveram iniciativas próprias, de acordo com suas capacidades. As desigualdades entre países, a diversidade de estratégias e as respostas nacionais autônomas influenciaram a atuação das organizações internacionais. A priorização dos governos de negociações bilaterais com farmacêuticas exacerbou a competição global por vacinas, enfraquecendo a cooperação internacional. No entanto, a diversidade de vacinas contratadas permitiu aos países enfrentar restrições de fornecimento e distribuição durante a pandemia. Nesse contexto, evidencia-se a necessidade de consolidar arranjos institucionais mais estáveis para a coordenação regional em futuras crises sanitárias, que reduzam a fragmentação das respostas e permitam maior alinhamento entre os governos. Ademais, a saúde deve ser colocada no centro dos sistemas sociais e econômicos.

A dependência tecnológica dos países constitui um ponto crucial para compreender as dificuldades e desafios das estratégias adotadas pela OMS e pela OPAS. Essa dependência é preocupante diante da crise do multilateralismo, que fragiliza o papel dessas organizações e compromete a capacidade da OMS de pautar a agenda global da saúde com autonomia e autoridade. A concentração da inovação e da produção reforça desigualdades e limita a efetividade das respostas multilaterais. O reforço da produção local e regional de vacinas, por meio de cooperação entre países, com apoio da OMS e da OPAS, pode reduzir a dependência da região em relação às importações, sendo uma estratégia crucial para o enfrentamento de futuras emergências sanitárias.

## Data Availability

As fontes de informação utilizadas no estudo estão indicadas no corpo do artigo.

## Supplementary Material

Material Suplementar
